# Association between industry payments and prescribing costly medications: an observational study using open payments and medicare part D data

**DOI:** 10.1186/s12913-018-3043-8

**Published:** 2018-04-02

**Authors:** Manvi Sharma, Aisha Vadhariya, Michael L. Johnson, Zachary A. Marcum, Holly M. Holmes

**Affiliations:** 1 Division of Geriatric and Palliative Medicine, McGovern Medical School, The University of Texas Health Science Center, Houston, TX USA; 2 Department of Pharmaceutical Health Outcomes and Policy, University of Houston, Houston, TX USA; 3 School of Pharmacy, University of Washington, Seattle, WA USA

**Keywords:** Open Payments, Sunshine Act, physician-industry financial relationship, Medicare Part D

## Abstract

**Background:**

While many new medications may offer advantages over existing drugs, some newer drugs are reformulations of existing products that provide little innovation or incremental benefit while driving up drug costs. Despite the lack of benefit of these medications, prescribers may be motivated by payments made by the pharmaceutical industry. The objective of the study was to determine the association between payments made to physicians by the pharmaceutical industry and prescriptions for certain selected costly brand name drugs.

**Methods:**

This was a cross-sectional, retrospective study linking the Open Payments Database and Medicare Part D Prescriber Public Use File for 2014, including 667,278 physicians who prescribed one of 6 brand-name drugs with less costly but similarly effective alternatives: lovastatin ER, almotriptan, amlodipine+olmesartan, ibuprofen+famotidine, saxagliptin+metformin and naproxen+esomeprazole. The primary outcome was the odds of a physician prescribing one of the selected drugs, and the primary predictor was the receipt of any payment from the pharmaceutical industry.

**Results:**

The odds of prescribing 3 of the 6 drugs were increased among physicians who received industry payment, compared to those without payment: amlodipine+olmesartan, aOR 1.42, (95% CI 1.36–1.49); saxagliptin+metformin, aOR 1.50, (95% CI 1.42–1.59); and naproxen+esomeprazole, aOR 1.45, (95% CI 1.25–1.68). Payment from the manufacturer of the specific drug, compared to not receiving payment from the drug’s manufacturer, was associated with increased odds of prescribing 4 of the 6 drugs: amlodipine+olmesartan, aOR 2.40, (95% CI 2.29–2.52), ibuprofen+famotidine, aOR 8.06, (95% CI 5.42–12.00), saxagliptin+metformin, aOR 2.21, (95% CI 2.10–2.34) and naproxen+esomeprazole, aOR 5.96, (95% CI 5.08–7.00).

**Conclusions:**

A physician-industry financial relationship was associated with increased odds of prescribing costly brand-name drugs of uncertain medical benefit. Patients, as healthcare consumers, should demand transparency from their physicians about payment from the pharmaceutical industry to increase shared decision-making. Physician and policy makers need increased awareness and reflection on how industry payment influences their prescribing practices.

## Background

Prescription costs in the U.S. are the highest in the world [1]. These high costs have been attributed not only to the complex drug development process but also to the restriction of price negotiation and government-protected monopolies granted to drug manufacturers [2]. Pharmaceutical manufacturers practice many methods to innovate and maintain market shares, including reformulating existing molecules and combining drugs [3]. While many new brand name drugs have clear medical benefits over alternatives already on the market, some do not, especially when considering the lower costs of existing alternatives. For example, Vimovo, a branded combination of naproxen and esomeprazole, has the potential benefit of improving tolerance of naproxen by co-administration with esomeprazole. However, with tolerance being the only clear benefit of the combined formulation, the listed wholesale acquisition cost (WAC) of $2259 for a bottle for 60 tablets^4^ may be hard to justify compared to the WAC of 60 tablets of naproxen ($6.36) and 60 tablets of esomeprazole ($225) [4].

The marketing of drugs to physicians includes various practices such as free drug samples, gifts, meals, speaker and consulting fees, and travel sponsorships. Because of the inherent risks for conflicts of interest and bias in physician-industry relations [5–9], policy makers have long called for increased transparency in these relations. The Physician Payments Sunshine Act requires that drug or device manufacturers report all payments made to physicians to the Centers for Medicare and Medicaid Services (CMS). CMS releases these reports in the Open Payments dataset (OPD) each year [10].

Most studies of physician-industry relationships have reported survey findings and qualitative data, until recent years. The release by CMS of the Medicare Part D Prescriber Public Use Files (MPDPUF) [11] has allowed studies on prescribing patterns of individual physicians to beneficiaries in the Medicare Part D system. Linkage of the OPD and the MPDPUF enables studies of the association between physician-industry relations and prescribing patterns using real-world data. Recently, studies using these linked datasets have found associations between industry payments and brand name prescribing [12, 13], regional patterns in prescribing of marketed drugs [14], and prescribing by ophthalmologists and the use of newer, more expensive medications [15].

While prior work has shown the presence of physician-industry relationships associated with prescribing, we aimed to expand this body of literature by focusing specifically on the prescribing of newer, more expensive drugs that offer little to no incremental clinical benefit to patients and adjusting for factors affecting the association between payments and prescribing. Thus, our objective was to determine the association between pharmaceutical industry payments to physicians and the physicians’ likelihood of prescribing costly brand name drugs for which there are similarly effective, less costly alternatives – medications that we defined as drugs of uncertain medical benefit. Our hypothesis was that payment from the pharmaceutical industry would be associated with greater likelihood of prescriptions of drugs of uncertain medical benefit.

## Methods

### Study Design & Data Sources

This retrospective, cross sectional study linked two publicly available datasets for 2014: the Open Payment Database (OPD) General Payments [10], and the Medicare Part D Prescriber Public Use Files (MPDPUF) [11].

OPD contains information on financial payments made by pharmaceutical and medical device companies to physicians and teaching hospitals. OPD is a national disclosure program mandated by the Affordable Care Act (ACA) and managed by the CMS. The types of payments made to physicians include consulting fees, research grants, travel reimbursements, meals, speaking fees, gifts, and other payments made from the industry to medical practitioners. OPD identifies physicians and teaching hospitals by name and address. The dataset contains the total value of payments to a particular recipient, the company who made the payment, and, if applicable, up to five covered drugs for which payment was attributed [16].

MPDPUF captures data on prescription drugs prescribed by physicians and other health care providers to Medicare Part D beneficiaries. MPDPUF identifies providers by their National Provider Identifier (NPI) [17]. For each prescriber and medication, the dataset includes the brand and generic name, the total days’ supply prescribed by that provider (which includes original and refill prescriptions) as well as the total drug cost based on the total amount paid by the Part D plan, Medicare beneficiary, government subsidies, and any other third-party payers. There is no individual-level beneficiary medication use data in the dataset [18].

Due to the absence of a common variable, a two-step process linked OPD with MPDPUF. First, OPD was linked to National Provider Identification database [17] based on the physicians first and last name, city and state. Then MPDPUF was linked using the common variable NPI. Physician records with missing name, city or state could not be merged and were excluded. Prescriber groups that did not have prescriptive authority or were not eligible for payments from the pharmaceutical industry (e.g., nurse practitioners, physician assistants, and pharmacists) also were excluded. The final analytic file included physician name, gender, address, city, state, zip code, physician specialty, drug name, total drug cost, total days’ supply for the drug, total amount of payments received, and amount of payment received by individual manufacturers.

**Fig. 1:**
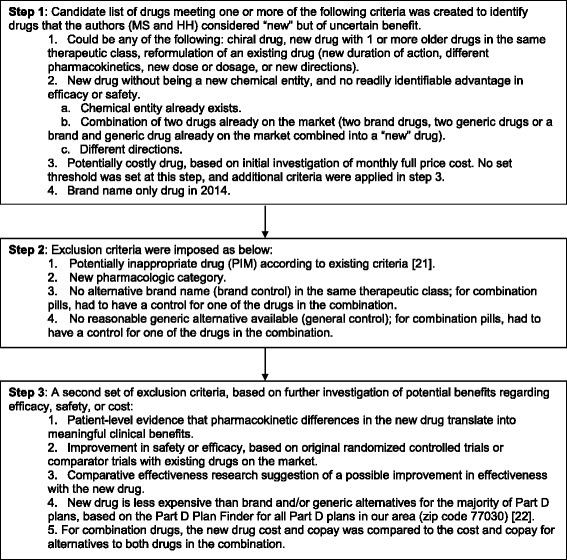
Selection of drugs of uncertain medical benefit

Two of us (HMH and MS) developed a candidate list of drugs based on use for common conditions, high cost, lack of benefit compared to other therapies in the same therapeutic category, and presence of similarly effective, less expensive therapies. These co-authors applied *a priori* criteria based on their clinical experience and the approved drugs database by the Federal Drug Administration (FDA) [4, 19, 20] to determine a list of drugs of uncertain medical benefit. Figure 1 describes the steps to select these drugs for the study [21, 22]. After the inclusions and exclusions, the list contained 7 drugs of uncertain medical benefit. An additional exclusion was imposed for Bromday (bromfenac ophthalmic solution) after discovering that it had been removed from the market. For each drug, a brand name drug and a generic drug in the same pharmacologic category were chosen to serve as “controls” for assessing the association between payment and prescribing. Each drug along with its brand and generic control were considered as a group and analyzed together. Table 1 shows the final list of drug groupings.

**Table 1:** Characteristics and utilization of drugs of uncertain medical benefit

Drugs	Approved Indication	Total Days Supply prescribed in 2014^a^	Total Drug Cost in 2014^b^	Cost/day (Total drug cost/total days’ supply)	AlternativesBrand Alternative Generic Alternative
Altoprev (Lovastatin ER)	Hyperlipidemia	32,954	512,685	15.56	Rosuvastatin (Crestor)
					Lovastatin
Axert (Almotriptan)	Migraine	20,433	546,958	26.77	Rizatriptan (Maxalt)
					Sumatriptan
Azor (Amlodipine+Olmesartan)	Hypertension	11,372,402	64,391,400	5.66	Telmisartan (Micardis)
					Irbesartan
Duexis (Ibuprofen+Famotidine)	Arthritis	76,607	1,994,159	26.03	Celecoxib (Celebrex)
					Ibuprofen
Kombiglyze XR (Saxagliptin+Metformin)	Diabetes	4,191,109	39,075,907	9.32	Sitagliptin (Januvia)
					Metformin
Vimovo (Naproxen+Esomeprazole)	Arthritis	518,280	13,104,735	25.29	Celecoxib (Celebrex)
					Naproxen

^a^Total days supply for the selected drug in Medicare Provider Utilization and Payment Data: Part D Prescriber Public Use File,

^b^Total cost for the selected drug per Medicare Provider Utilization and Payment Data: Part D Prescriber Public Use File

### Ethical considerations

The Institutional Review Board (IRB) of the University of Houston determined the study to be exempt.

### Outcome measures and predictors

The primary outcome of interest was whether a physician prescribed a drug of uncertain medical benefit, defined as a dichotomous (yes/no) variable for any prescribing of the drug (days’ supply > 0) vs. not prescribing it. The primary predictor of interest was payment from the pharmaceutical industry, defined as a dichotomous (yes/no) variable for receipt of any payment. Covariables included gender, specialty (general practice, internal medicine specialties, others), census region (Northeast, Midwest, South and West), overall physician volume of prescribing and prescribing volume in the therapeutic category. The therapeutic category prescribing volume was calculated as the days’ supply of all prescriptions for any drug within the same therapeutic category as the drug of uncertain medical benefit. The overall prescribing volume of a physician was calculated as the total days’ supply of all drugs of any category prescribed by that physician. A log of overall prescribing volume of a physician and the therapeutic category prescribing volume were used for improved model specification [23]. Secondary analyses tested the association of payment from the manufacturer of the selected drug with the primary outcome.

### Statistical Analysis

All statistical analyses were performed using SAS 9.3 (SAS Institute, NC). Each drug of uncertain medical benefit and the corresponding brand and generic controls formed a trio group consisting of physicians who prescribed at least one of the three drugs. Thus, each drug in the trio had the same number of physicians analyzed, and each trio had a unique physician sample. Bivariate analysis compared demographic characteristics of physicians who were ever paid vs. never paid in the OPD. Univariate logistic regression analysis tested the association between industry payments and prescription of drugs of uncertain medical benefit. Multivariable logistic regression tested the association of receiving any industry payment versus not as the primary predictor of interest with prescribing the drug of uncertain medical benefit versus not as the primary outcome, controlling for physician gender, specialty, region, therapeutic category prescribing and overall prescribing volume. Similar separate models tested each drug of uncertain medical benefit as well as each brand name control and each generic control in each trio group. All analyses were performed at an *a priori* alpha level of 0.05.

## Results

**Fig. 2:**
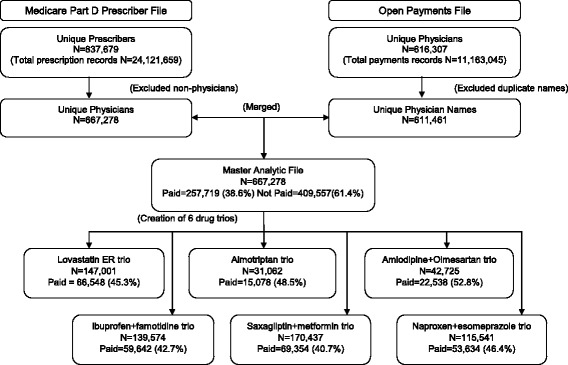
Study selection flow diagram

From 837,679 individual prescribers who prescribed drugs to Medicare Part D beneficiaries in 2014, 667,278 met inclusion and exclusion criteria, 38.6% (*n* = 257,719) of whom received at least one payment from the pharmaceutical industry. Figure 2 shows the selection of physicians included in the study, the number of physicians evaluated for each drug of uncertain medical benefit, alternative brand, and alternative generic, and the percent of physicians who received any payment for each trio. Among prescribers of the drugs of uncertain medical benefit, 58% to 63% received at least one payment. Among prescribers of brand control and generic alternative drugs, 48% to 59% and 39% to 51%, respectively, received at least one payment. The total drug cost and total days’ supply for the selected drugs of uncertain benefit for Part D beneficiaries from the MPDPUF data for 2014 is shown in Table 1. The range of cost for the selected drugs of uncertain medical benefit were from $5 to $26 per day [4]. The summary of number and percent of prescribers that received payments for each drug are presented in Table 2.

**Table 2:** Number of prescribers for each drug and number of prescribers that received payments

S.No.	Drug Name	Number of prescribers of the drug^a^ N	Prescribers that received any payments^b^ N (%)
1.	Lovastatin ER	81	47 (58.02%)
	Rosuvastatin	125,058	60,192 (48.13%)
	Lovastatin	96,189	42,631 (44.32%)
2	Almotriptan	87	53 (60.92%)
	Rizatriptan	224	115 (51.34%)
	Sumatriptan	30,940	15,012 (48.53%)
3	Amlodipine+olmesartan	11,018	6687 (60.69%)
	Telmisartan	2152	1273 (59.15%)
	Irbesartan	35,546	18,308 (51.51%)
4	Ibuprofen+famotidine	116	66 (56.90%)
	Celecoxib	75,422	39,328 (52.14%)
	Ibuprofen	104,759	41,530 (39.64%)
5	Saxagliptin+metformin	5265	3122 (59.30%)
	Sitagliptin	82,593	40,227 (48.71%)
	Metformin	168,819	68,718 (40.70%)
6	Naproxen+esomeprazole	787	498 (63.28%)
	Celecoxib	75,422	39,328 (52.14%)
	Naproxen	79,189	35,057 (44.27%)

^a^The number of prescribers in each trio are not mutually exclusive, i.e. same prescriber could prescribe either 1, 2 or all 3 drugs in the trio

^b^The number (and percent) of prescribers that received any payment from industry is among the prescribers of the specific drug

Table 3 shows the multivariable analysis for the odds of prescribing each drug by physicians who received any payment vs. those who did not. Any industry payment was associated with increased odds of prescribing of all the drugs of uncertain medical benefit, with non-significant findings for lovastatin ER, almotriptan and ibuprofen+famotidine. Payment was associated with increased odds of prescribing of all brand name controls except for rizatriptan. For all generic controls, payment was associated with a decrease in the odds of prescribing of the generic drug, with non-significant findings for sumatriptan.

**Table 3:** Adjusted odds ratios^a^ (aORs) and 95% confidence intervals (CIs) for prescribing vs. not prescribing a drug based on receiving any payment vs. no payment^b^

Drug of Uncertain Medical Benefit	aOR (95%CI)	Brand Control	aOR (95%CI)	Generic Control	aOR (95%CI)
Lovastatin ER	1.28 (0.82–2.80)	Rosuvastatin	**1.80 (1.74–1.86)**	Lovastatin	**0.78 (0.76–0.80)**
Almotriptan	1.36 (0.83–2.23)	Rizatriptan	0.93 (0.68–1.27)	Sumatriptan	0.74 (0.50–1.10)
Amlodipine+olmesartan	**1.42 (1.36–1.49)**	Telmisartan	**1.18 (1.08–1.29)**	Irbesartan	**0.74 (0.70–0.78)**
Ibuprofen+famotidine	1.10 (0.76–1.60)	Celecoxib	**1.85 (1.80–1.89)**	Ibuprofen	**0.79 (0.77–0.81)**
Saxagliptin+metformin	**1.50 (1.42–1.59)**	Sitagliptin	**1.45 (1.42–1.48)**	Metformin	**0.80 (0.72–0.89)**
Naproxen+esomeprazole	**1.45 (1.25–1.68)**	Celecoxib	**1.61 (1.57–1.65)**	Naproxen	**0.74 (0.72–0.76)**

^a^Adjusted for gender, specialty, region, therapeutic category and overall prescribing volume

^b^Each drug was analyzed in a separate model

*p*-value <0.05 are presented in boldface

Table 4 presents the odds ratios for prescribing the selected drugs by physicians with payments from the individual drug manufacturer. Payment by the drug manufacturer was associated with increased odds of prescribing of the drug manufactured by that company for all drugs except lovastatin ER and almotriptan.

**Table 4:** Adjusted odds ratios and 95% confidence intervals for prescribing vs. not prescribing a drug based on receiving any payment vs. no payment from the manufacturer making the drug^a^

Drug	Manufacturer	Odds Ratio	95% Confidence Interval
Lovastatin ER	Actavis (Andrx)	2.59	0.93–7.18
Almotriptan	Janssen	1.40	0.75–2.61
Amlodipine+olmesartan	Daiichi-Sankyo	**2.40**	**2.29–2.52**
Ibuprofen+famotidine	Horizon	**8.06**	**5.42–12.00**
Saxagliptin+metformin	AstraZeneca	**2.21**	**2.10–2.34**
Naproxen+esomeprazole	Horizon	**5.96**	**5.08–7.00**

^a^adjusted for gender, specialty, region, therapeutic category and overall prescribing volume

*p*-value <0.05 are presented in boldface

## Discussion

This study of 667,278 physicians prescribing to Medicare Part D beneficiaries found that receiving any payment from the pharmaceutical industry was associated with increased odds of prescribing drugs of uncertain medical benefit. Brand name alternatives were also more likely to be prescribed when physicians received any industry payment. There was a pattern of lower likelihood of prescribing the generic alternative with receipt of any payment. While our study findings are consistent with prior studies showing the relationship between pharmaceutical industry payments and the prescription of brand name drugs [14], this study evaluates the prescription of selected expensive drugs that add no apparent additional benefit to a market already full of alternatives. This study adds to the literature by finding that this association is consistent in direction and magnitude for new drugs of uncertain medical benefit while adjusting for multiple factors including physician specialty and prescribing volume. The magnitude of the association between prescription and payments was even higher when the payment from the specific manufacturer was evaluated with prescription for that drug.

This study evaluated those drugs that were of the least discernible benefit in light of significant increases in costs. The drugs chosen in this study were combination drugs, in which convenient dosing and adherence might be the only benefits, namely, Kombiglyze XR and Azor. Also included were long-acting formulations of existing drugs (Altoprev) and newer drugs structurally similar to existing drugs (Axert). Finally, as is the case with Duexis and Vimovo, this study included drugs available without prescription at negligible cost that are marketed as combination branded drugs, costing more than $2000 per month in 2016 [24].

Our findings are consistent with other recent studies using the OPD and MPDPUF data [12, 13, 25, 26]. Yeh et al. found that pharmaceutical payments were associated with higher rates of prescribing brand name statins [12]. Perlis et al. found an association between payments and brand name prescribing as well as total prescription costs in the Medicare Part D program, an association that was found across multiple physician specialties [13]. Moreover, Fleischman et al. found an association between pharmaceutical payments and regional prescribing of “marketed” drugs – drugs for which there were 100 or more payments made by industry between August 2013 and December 2014 – in the Part D program [14]. DeJong et al. found a significant association between brand name prescribing and a corresponding industry-sponsored meal for the drug [27]. Qian et al. reported a decreased rate of generic drug prescribing with receipt of payments [28]. Finally, Propublica reported findings of their analysis of the OPD and MPDPUF data, showing that payment was associated with brand name prescribing [26]. It is clear from the published literature that payment from the pharmaceutical industry impacts prescribing practices.

The objective of our study was different than prior studies evaluating the association of industry payment and prescribing as we evaluated select drugs that we deemed to be of uncertain medical benefit based on criteria set *a priori* to identify medications that are newer, more expensive formulations of drugs for which reasonably equivalent, less expensive drugs would be a better choice. If a hypothesized relationship between payments and drug prescribing exists, we expected to detect this by comparing such new and expensive drugs with well-known generic alternatives. Our findings supported this hypothesis for three out of the six drugs selected – amlodipine+olmesartan, saxagliptin+metformin and naproxen+esomeprazole. For the remaining three drugs – lovastatin ER, almotriptan and ibuprofen+famotidine, there were considerably fewer physicians prescribing them, and thus we may not have had an adequate sample size to detect a statistically significant association.

The major strength of this study was the focus on expensive, brand name drugs for which there are reasonable alternatives and uncertain medical benefit. Our interest was in evaluating drugs whose purpose based on our inclusion/exclusion criteria was more related to gaining market share without meaningful innovation. These drugs were priced higher than alternatives available in the market at the same time and may have been promoted to physicians with financial incentives. An additional strength of our study was the adjustment for multiple potential confounders. In our analysis, we found that all bivariate associations for available covariates in the datasets were consistently significantly associated with prescribing of drugs of interest. We adjusted for all covariates in multivariable analysis, and notably found that controlling for physician specialty and therapeutic category volume markedly decreased the apparent effect size of the association between payment and prescribing. Another advantage of our study was the use of publicly available datasets that included 99.9% of Medicare Part D beneficiaries, enhancing generalizability. Lastly, a significant strength of our study is the analysis of the association between payments from specific manufacturer of the selected brand name drugs and prescribing of the selected brand name drugs.

### Limitations of the study

Our study has a few important limitations. The linking of the two datasets could have underestimated the number of prescribers included in the dataset; however, our results, including the prevalence of payment to physicians, were similar to those found in other similar studies using the same datasets. If we underestimated the number of prescribers in the dataset, we have no reason to believe that the underestimation would have occurred in a biased fashion. This was a cross-sectional study, thus we cannot assert causation between payments and prescribing. Also, since there was no patient-level data, we cannot determine if small groups of patients drove this association. Physicians may have chosen the drugs for valid reasons that may be patient-centered, such as improved adherence, but we could not adjust for any patient-level factors or clinical decision making rationale. We also excluded nurse practitioners and physician assistants who may prescribe under the direction of physicians who received payment. Future research is needed to evaluate the impact of industry payments on prescribing among clinics where multiple provider types are practicing.

## Conclusions

Our study adds to the accumulating evidence of the influence of pharmaceutical industry payments on the use of newer, expensive brand name medications that have similarly effective, less costly alternatives. Given the possible conflict of interest inherent in physicians receiving pharmaceutical payments, the transparency of the data and the availability of tools such as Dollars for Docs [29] will make it easier for patients and consumers to understand the possible influence of the pharmaceutical industry on a physician’s decision to use a newer, more expensive drug, including drugs of uncertain medical benefit [30]. There may be a perception among the public that a new drug, and its implied novelty, works better than existing drugs in the same class. However, using evidence-guided criteria, our study identified drugs where this may not be the case and found a significant association between pharmaceutical industry payment and physician prescribing of these drugs. These findings contribute to growing evidence of the role of pharmaceutical industry payment in prescribing practice. Patients could use these findings to be more informed as healthcare consumers in order to advocate for shared decision-making at the time of prescribing new drugs. Providers could use these findings to be more aware of the explicit or implicit effects of receiving payments on their prescribing practices. Policymakers could use these findings to develop policies to improve interactions between the pharmaceutical industry and providers in order to improve the quality of care and reduce drug costs.

## Abbreviations

ACA: Affordable Care Act
CMS: Centers for Medicare and Medicaid Services
FDA: Federal Drug Administration
IRB: Institutional Review Board
MPDPUF: Medicare Part D Prescriber Public Use Files
NPI: National Provider Identifier
OPD: Open payments dataset
WAC: Wholesale acquisition cost
